# Conifer By-Products Extracted Using Hydrodynamic Cavitation as a Convenient Source of Phenolic Compounds and Free Amino Acids with Antioxidant and Antimicrobial Properties

**DOI:** 10.3390/molecules30132722

**Published:** 2025-06-25

**Authors:** Luisa Pozzo, Andrea Raffaelli, Lidia Ciccone, Federica Zabini, Andrea Vornoli, Vincenzo Calderone, Lara Testai, Francesco Meneguzzo

**Affiliations:** 1 Institute of Agricultural Biology and Biotechnology, National Research Council of Italy, Via Moruzzi 1, 56121 Pisa, Italy; luisa.pozzo@cnr.it (L.P.); andrea1.raffaelli@santannapisa.it (A.R.); andrea.vornoli@cnr.it (A.V.); 2 Crop Science Research Center, Scuola Superiore Sant’Anna, Piazza Martiri della Libertà 33, 56127 Pisa, Italy; 3 Department of Pharmacy, University of Pisa, Via Bonanno, 56126 Pisa, Italy; lidia.ciccone@unipi.it (L.C.); vincenzo.calderone@unipi.it (V.C.); 4 Interdepartmental Center Nutrafood “Nutraceuticals and Food for Health”, University of Pisa, 56124 Pisa, Italy; federica.zabini@cnr.it; 5 Institute of Bioeconomy, National Research Council of Italy, Via Madonna del Piano 10, 50019 Firenze, Italy; francesco.meneguzzo@cnr.it

**Keywords:** conifers, specialized metabolites, biological activities, waste, sustainable extraction

## Abstract

Softwood bark and twigs represent by-products of forest supply chains rich in extractable bioactive compounds. This study aimed at evaluating the bioactive molecules of hydrodynamic cavitation (HC) based extracts of bark and twigs from different conifer plants and exploring their antioxidant capacity. Samples of *Picea abies* twigs (RAR) and bark (CAR) and *Abies alba* twigs (SFT) underwent extraction using a pilot-scale Venturi reactor HC device. The freeze-dried extracts were characterized for the antioxidant capacity, through both in vitro and ex vivo assays, the antimicrobial activity, and the content of phenolics and free amino acids by UHPLC-ESI-MS/MS. HC-based aqueous extracts were obtained quickly and with low energy consumption. We found 10 phenolic acids, nine flavonols, three flavan-3-ols, five flavanones, three procyanidins, two stilbenoids, and 10 other phenolic compounds. Moreover, eight essential and seven dispensable amino acids were found. The principal component analysis showed clear discrimination among the three extracts. The CAR extract showed antimicrobial activity. The SFT extract showed the higher anthocyanins content and antioxidant activity, both through in vitro and ex vivo methods. These preliminary results confirm that by-products of *Picea abies* and *Abies alba* are rich in bioactive compounds and antioxidant activities, suggesting potential applications in the nutraceutical and pharmaceutical fields.

## 1. Introduction

Plant biomass, especially from conifers, is rich in chemicals, with potential use in various fields, from pharmaceuticals and food to green polymers and bio-based materials [1,2,3]. Softwood barks, which represent—like twigs—by-products of the forest-wood supply chains, contain high levels of extractable compounds, including a whole range of specialized metabolites such as valuable polyphenols and tannins, mostly condensed and hydrolysable [4]. Conifer biomass contains valuable phytochemicals with therapeutic potential [5]. Numerous studies have highlighted the health benefits of pine bark extracts. In particular, supplementations with conifer extracts improved cardiometabolic and cardiovascular risks, suggesting the possibility of an adjunct therapeutic strategy for the management of metabolic disorders. Furthermore, neuroprotective effects have been demonstrated with pine bark extracts in transient forebrain ischemia models and under other experimental conditions of cognitive decline. Finally, anti-ageing effects have been also observed. The broad spectrum of benefits has been assumed to be due—at least in part—to antioxidant and anti-inflammatory activities [6,7,8]. Therefore, the evaluation of antioxidant potency is currently a useful preliminary assay in order to hypothesize the putative uses of conifer extracts.

Interestingly, supplements, cosmetics and foods based on pine bark are already commercially available (e.g., Pycnogenol and Enzogenol) [9,10]. Together with pine, Norway spruce and silver fir are the most abundant coniferous species in Eurasian forests, including the Italian Alps and, to a lesser extent, the Northern Apennines.

Highly bioactive compounds, such as glycosylated monomeric stilbenes, astringin, taxifolin, piceid and isorhapontin [11,12], dimeric stilbenes with astringin and astringin-isorhapontin dimers [13], hydroxystilbenes, resveratrol, isorhapontigenin and piceatannol acetate derivatives have been identified in spruce bark and needles [14,15].

The recovery and valorization of phenolic compounds from biomass of forest by-products, in particular, from the forest-wood supply chains, is attracting considerable attention [16,17,18], from a scientific, industrial and political-social point of view, also with a view to repopulating the internal areas of the country. In this sense, two of the most important elements are the sustainability of the extraction methods and the effectiveness in the use of the products obtained. The fundamental barrier, which hinders the efficient valorization of the precious by-products of the conifer forest supply chains, consists in the unavailability of efficient extraction techniques. Conventional extraction techniques, such as hot water extraction or the use of organic solvents, present significant limitations in terms of environmental impact and energy and resource consumption, adding to limited yield [19]. For this reason, research has focused for several years on identifying alternative, more efficient and less impactful extraction techniques. Among the unconventional methods applied to the extraction of phenolic compounds from coniferous parts, we recall ultrasound-assisted extraction (UAE) [20], microwave-assisted extraction (MAE) [21], pressurized liquid extraction (PLE) [19], extraction with supercritical fluids [22], enzymatic extraction [23] and hydrodynamic cavitation (HC) extraction [24].

HC is emerging as one of the most promising and innovative technologies for the extraction of natural products from whole plant materials, including by-products of the supply and processing chains. Cavitation is a physical-chemical phenomenon of formation, growth and implosion of vapor bubbles in a liquid at temperatures below boiling point, which generates microenvironments characterized by locally very high temperatures, intense pressure waves and hydraulic jets, and excess generation of oxidizing radicals, capable of intensifying a series of physical, chemical and biochemical processes in an efficient and “green” way. The absence of chemicals such as mineral acids, bases or organic solvents that potentially degrade or contaminate the extracted bioproducts ensures less product degradation and higher product quality for bioproducts extracted via cavitation technology. In particular, organic solvent-based methods require preventive removal of lipophilic compounds, for example, using Soxhlet methods and subsequent extraction steps using different solvents, such as n-hexane for stilbenoids and acid-ethanol for the recovery of pectin from spruce bark, leading to highly selective extraction and the need for expensive solvent recovery [25]. However, as pointed out and proven in a previous study [26], greener (water only) and cheaper processes, such as hot water or HC, not only avoid the solvent removal steps, but also afford integral extracts (including lipophilic and higher molecular weight polyphenols) showing broader-spectrum biological effects than purified substances, provided sufficient energy is delivered to biomass particles.

HC efficiency has already been verified on a full scale with numerous biological matrices, including malt and hops for beer production [27], citrus pulp and peel [28], silver fir needles [29], chestnut sawdust [30], and pomegranate [31]. HC offers distinct advantages, such as the use of water as the only process solvent; comparatively limited process times and temperatures; electricity as the only energy source; and straightforward scalability.

The focusing of extreme energy density into localized hot spots with consequent thermal, mechanical and chemical processes lies behind the comparatively higher intensification of chemical, physicochemical, and biochemical reactions, making HC a more efficient and cost-effective technology compared to other techniques, such as Soxhlet, microwave, and supercritical fluid extraction, in the extraction of plant resources and in several other applications [32]. In particular, HC was shown to outperform hot water in the extraction of *Picea abies* bark by more than 7 times [26], with HC-based extracts also showing higher antibacterial activity likely due to lower temperature processing and the higher retention of terpenoids.

The possibility of producing functional solutions without the use of any synthetic solvent, in a way that is not only rapid, economical and scalable up to the industrial level, but also capable of operating with high extraction yields, represents a great opportunity for several sectors. Potential application fields for these extracts of forest by-products are the functionalization of food products, such as pasta and bakery products, to increase their nutritional value, as well as food supplements, cosmeceutical products and prospectively even pharmaceutical products.

## 2. Results and Discussion

### 2.1. Extraction of Conifer Resources

**Figure 1:**
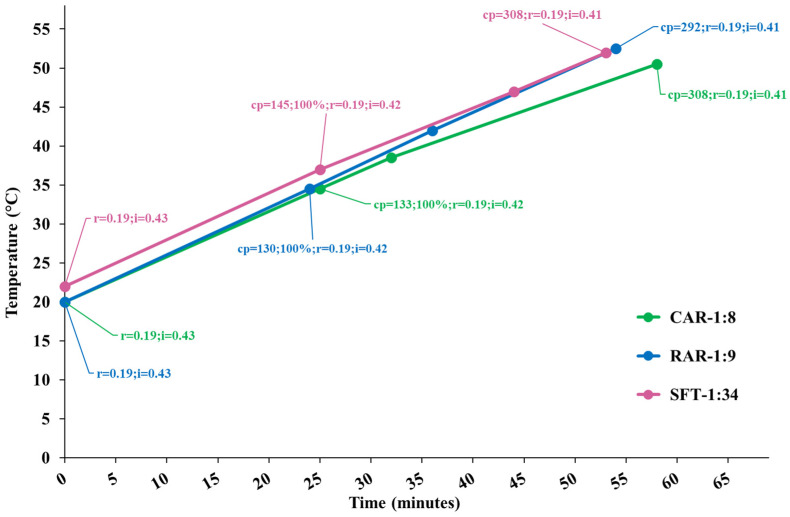
Temperature diagram as a function of the process time for extraction runs CAR, RAR and SFT. Data tags show the levels of the cavitation number in the static HC reactor (r) and pump’s impeller (i) zones, cavitation passes (cp) and the moment of 100% insertion of biomass.

Figure 1 shows the temperature diagram as a function of the process time for the performed extractions, along with the cavitation number in the static HC reactor and at the pump impeller and the cavitation passes. Processes were very similar, and developed cavitation regimes occurred in both cavitation zones for all the processes, with more intense cavitation (lower cavitation number) in the static reactor. The extracts were sampled at the end of each process.

### 2.2. Total Extraction Yield

Table 1 shows the experimental results of the HC extraction in terms of the total extraction yield and total phenols in the freeze-dried extract. The extraction yield was significantly affected by the specific processed resource. The RAR extract showed a significantly higher yield (164.55 mg/g dw), followed by CAR (141.97 mg/g dw) and SFT (135.91 mg/g dw). These figures, which can be useful for the purposes of a technical-economic assessment, outperformed the extraction yields obtained by Spinelli and colleagues [19] with a supercritical fluid extraction (SFE) at different ethanol concentrations (10–70%) from the bark of Norway spruce, where they found levels between 28.6 and 31.2 mg/g dw depending on the percentage of ethanol used for extraction. In the same study, higher extraction yields were reported using pressurized liquid extraction (PLE) with water (130.7 mg/g dw) and with ethanol (127.9 mg/g dw) and through UAE with 70% ethanol (123.3 mg/g dw) from bark of Norway spruce, which were closer to the corresponding yield achieved using HC (141.97 ± 8.47 mg/g dw).

### 2.3. Phytochemical Composition of Conifer Extracts

The assay with Folin–Ciocalteu’s reagent was carried out to determine total phenols content of the extract samples. The results, expressed as mg of gallic acid equivalent (GAE) per g, are shown in Table 2. Total phenols content ranged from 75.71 to 108.11 mg GAE/g. RAR extract showed a significantly lower total phenolic content compared to SFT and CAR extracts. Total flavonoids content was expressed based on mg of catechin equivalent (CE) per g and varied from 59.43 to 89.18 mg CE/g (Table 2). Also, RAR extract showed a significantly lower content of flavonoids compared to SFT and CAR extracts. The total flavonols were expressed as mg of quercetin equivalent (QE) per g and ranged between 4.16 mg QE/g in RAR extract and 6.84 and 6.08 mg QE/g in CAR and SFT extracts, respectively, which showed a significantly higher content (Table 2). The total anthocyanins were expressed as µg of cyanidin-3-glucoside equivalent (C3GE) per g and ranged between 0.35 µg C3GE/g in RAR extract and 8.23 µg C3GE/g in SFT extract, which showed a significantly higher content compared to RAR and CAR (Table 2).

Significant variability exists in the yields of secondary phenolic metabolites across tree species, within different tree parts, and even among individuals of varying ages and botanical regions of provenance [33]. In a previous study by Kähkönen et al. [34], a total phenolic concentration of 76.0 mg GAE/g was found for dried bark of Scots pine (*P. sylvestris*), equal to that observed in the present study in RAR, but lower than those seen in CAR and SFT.

A study by Nisca et al. [21] aimed to evaluate and compare the phytochemical profiles of hydroalcoholic *Picea abies* bark extracts obtained via UAE and MAE, as well as their antioxidant and antibacterial activities. The results showed that UAE extracts contained higher levels of total polyphenols, tannins, and key phenolic compounds—particularly catechin and epicatechin—compared to MAE extracts, although overall phenolic yields were lower than those obtained in our study (all values approximately 40 mg GAE/g or lower).

In a study conducted by Schoss et al. [35] on six *Picea abies* branches collected in Slovenia, a mean phenolic content of 28.7 mg GAE/g was reported in samples comprising both wood and bark, resulting in a value far lower than that found in our study for twigs of the same species. Interestingly, each branch was segmented at regular 10 cm intervals up to 80 cm from the trunk, revealing a marked decrease in phenolic content with increasing distance. Specifically, the segment at 80 cm exhibited approximately 61% lower phenolic levels compared to the segment at the trunk base. These findings highlight significant spatial variation in phenolic distribution. Based on these findings, the notably higher phenolic contents observed in the twigs are particularly remarkable, underscoring the enhanced efficiency of our extraction approach.

The phenolic compound profile of the extracts (Table 3) was determined by liquid chromatography–tandem mass spectrometry (LC–MS/MS). In total, 42 compounds were detected in each extract.

SFT extract showed significantly (*p* < 0.05) higher contents in gallic acid (274.30 µg/g), protocatechuic acid (357.22 µg/g), rosmarinic acid (0.14 µg/g), vanillic acid (82.09 µg/g), p-coumaric acid (142.21 µg/g), ∑ phenolic acids (908.39 µg/g), (+)-catechin (97.14 µg/g), (−)-epicatechin (7.13 µg/g), ∑ flavan-3-ols (104.27 µg/g), naringenin (3.42 µg/g), eriodictyol (1.09 µg/g), ∑ flavanones (4.88 µg/g), hydroxytyrosol (4.91 µg/g), verbascoside (0.33 µg/g), pinosterol (37.76 µg/g) and phloretin (0.20 µg/g) compared to the other two extracts. Gallic acid and protocatechuic acid were the most abundant; these are frequently reported in Pinus species bark, with protocatechuic acid acting as a degradation product of complex tannins [36]. Catechin and epicatechin are consistent with flavan-3-ols found in pine bark extracts, where total catechin-type compounds can exceed 1000 µg/g in some commercial products [37]. However, taxifolin (dihydroquercetin), a key bioactive in pine bark (especially Larix and Pinus spp.), was not reported or detected in this HC extract. This may be due to the lower abundance in the specific plant matrices used, the instability or degradation during HC processing or the lack of LC–MS/MS standard or detection limits.

The highest levels observed in RAR were instead those of chlorogenic acid (1.08 µg/g), chicoric acid (4.58 µg/g), quercetin (0.58 µg/g), quercetin 3-O-glucoside (4.33 µg/g), rutin (16.49 µg/g), quercetin 3,4-O-diglucoside (16.30 µg/g), quercetagetin 3-O-glucoside (5.59 µg/g), kaempferol 7-*O*-glucoside (17.69 µg/g), kaempferol 3-*O*-glucoside (107.28 µg/g), tiliroside (1.24 µg/g), kaempferol 4-*O*-glucoside (34.91 µg/g), ∑ flavonols (204.41 µg/g), naringin (0.29 µg/g), procyanidin B1 (78.15 µg/g), procyanidin B2 (35.69 µg/g), procyanidin B3 (14.69 µg/g), ∑ procyanidins (128.80 µg/g), piceid (68.06 µg/g), resveratrol (0.56 µg/g), ∑ stilbenoids (68.62 µg/g), oleuropein (0.13 µg/g), ligstroside (0.12 µg/g), phloridzn (1.56 µg/g), luteolin (0.06 µg/g) and apigenin (0.05 µg/g). The RAR extract exhibited a remarkably high flavonol content of 204.41 µg/g, predominantly comprising kaempferol 3-*O*-glucoside, rutin, and quercetin 3,4-O-diglucoside. These flavonol glycosides are more commonly associated with leaf and floral tissues of angiosperms and are rarely dominant in gymnosperm bark [38]. In addition to flavonols, the RAR extract contained notable levels of stilbenoids, particularly piceid and resveratrol, compounds that are typically abundant in grapevine (*Vitis vinifera*) and deciduous hardwood species [39]. While stilbenes can occur in conifer bark, their presence is usually limited to trace levels and is often associated with environmental stressors such as UV radiation or pathogen exposure [40]. The overall polyphenolic profile of RAR therefore appears to resemble that of deciduous hardwoods more than the typical profiles observed in conifer extracts, which are generally characterized by proanthocyanidins and catechins [37]. However, the detection of quercetin glycosides and procyanidins in RAR also reflects components that, while minor, are known to occur in conifer bark under certain conditions. This suggests that the RAR extract either originates from a botanically diverse source, includes non-woody tissues, or reflects stress-induced metabolic shifts that broaden its phenolic composition.

Finally, CAR extract showed a significantly (*p* < 0.05) higher content of protocatechuic acid (348.52 µg/g), caffeic acid (20.40 µg/g), *trans*-ferulic acid (135.33 µg/g), cynarin (0.03 µg/g), hesperetin (0.12 µg/g), hydroxytyrosol a-acetate (0.63 µg/g), pinosterol (39.06 µg/g) and luteolin (0.08 µg/g). A high content of lignin-related phenolic acids was observed, particularly in trans-ferulic acid and caffeic acid, both of which are key intermediates in lignin biosynthesis and are commonly associated with the structural integrity of woody tissues, especially within the Pinaceae family [41]. In addition to these hydroxycinnamic acids, CAR also contained a notable concentration of pinosterol, a phytosterol reported in various pine tissues but less frequently characterized in bark-specific phytochemical studies [42]. Furthermore, the detection of low yet significant levels of flavones, such as luteolin and apigenin, suggests minor contributions from photosynthetic tissues like needles, as these flavones are more typical of foliar phenolic profiles and are rarely dominant in conifer bark [43]. Collectively, the CAR extract appears to reflect a phenolic profile skewed toward lignin-derived and structural metabolites, indicative of a higher proportion of mature, protective woody tissues. This composition supports the hypothesis that CAR is enriched in compounds related to plant defense and mechanical stability rather than in antioxidant-rich polyphenols more typical of reproductive or stress-induced tissues.

The free amino acidic profile of the extracts (Table 4) was determined by liquid chromatography–tandem mass spectrometry (LC–MS/MS). In total, 15 compounds were detected in each extract.

All the essential amino acids (valine, threonine, leucine, isoleucine, leucine/isoleucine, lysine, methionine + cystine, phenylalanine + tyrosine and histidine) showed significantly (*p* < 0.05) higher content in SFT extract compared to RAR and CAR extracts.

Regarding the dispensable amino acids, among the three extracts analyzed, SFT extract showed the highest (*p* < 0.05) content in glycine (12.24 ng/g), serine (22.08 ng/g) and glutamin acid (164.84 ng/g), while the RAR extract showed the highest content in aspartic acid (39.67 ng/g), and the CAR extract the highest content of alanine (95.20 ng/g) and proline (77.27 ng/g).

Although conifer bark is traditionally studied for its polyphenolic and tannin-rich composition, its potential as a source of free amino acids has received limited attention. Notably, the SFT extract demonstrated exceptionally high levels of essential amino acids, particularly leucine and threonine. These concentrations significantly exceed typical values reported for pine bark hydrolysates, which are generally dominated by non-essential amino acids and protein-bound nitrogen fractions [44]. The enhanced recovery of these bioavailable amino acids in the SFT extract suggests that the HC method may facilitate more efficient release of soluble nitrogenous compounds compared to conventional extraction approaches. This observation expands the known bioactive profile of conifer bark beyond polyphenols and supports its underexplored nutritional potential. Specifically, SFT’s amino acid richness may offer value in applications aiming to recover both antioxidant and nutritional components from forestry by-products.

### 2.4. Antioxidant Properties

The results of the antioxidant activity analyses of the extracts, determined as ferric-reducing antioxidant power (FRAP), DPPH scavenging activity, and oxygen radical absorbance capacity (ORAC), are shown in Table 5. The range of FRAP values of the samples was between 104.71 and 175.65 mg TE/g, and SFT extract showed the significantly highest result followed by CAR extract and RAR extract.

The results for DPPH scavenging activity showed no significant differences between samples.

In turn, ORAC results were between 507.26 and 687.57 mg TE/g, and the ORAC was higher (*p* < 0.01) for SFT and CAR extracts compared to RAR extract.

Notable differences in antioxidant performance among the three extracts highlight the multifaceted nature of antioxidant mechanisms and the influence of extract composition and sampling strategy. The SFT extract exhibited the highest antioxidant capacity in both the FRAP and ORAC assays. These results suggest a higher concentration of compounds with strong electron-donating and hydrogen atom transfer (HAT) capabilities, respectively. Such activity is typically associated with elevated levels of total phenols and flavonoids, as corroborated by the compositional data presented in Table 2. Phenolic compounds, particularly those with ortho-dihydroxy substitutions on the aromatic ring, have been consistently shown to exhibit strong reducing power and free radical scavenging potential due to resonance stabilization of the resulting phenoxyl radicals [45,46]. The lack of strong correlation between DPPH activity and total phenolic content may reflect a compositional bias favoring compound classes with selective DPPH reactivity, such as flavonols and procyanidins, which were more abundant in the RAR extract (Table 3), as their multiple hydroxyl groups facilitate efficient hydrogen donation [47].

The number and position of the phenolic groups may influence the antioxidant activity, so different phenolic compounds reveal different biological functions [48]. Notably, the antioxidant activity according to the FRAP and ORAC assays was positively associated with total phenols and flavonoids shown in Table 2, while DPPH did not show such association. Based on Table 3, it is possible that the relatively high DPPH level shown by the RAR extract was due to the higher content of flavonols or procyanidins, the latter known as particularly effective for the scavenging of DPPH radicals [49].

In a study by Chmelova et al. [50], the efficiency of conventional solvent extraction (SE) and UAE of *Picea abies* bark was compared in terms of antioxidant capacity (AC) and total polyphenol content (TPC). In both cases, the values obtained in terms of both AC and TPC were lower than those observed in our study at all the tested conditions thanks to the improved extraction capacity of the technique we used.

The antioxidant activity of branch extracts declined with increasing distance from the trunk [35]. Extracts from segments located 80 cm away showed a 52% reduction in DPPH scavenging capacity (from 12.2 ± 1.1 to 5.8 ± 2.8 mg GAE/g) and a 51% reduction in ABTS radical cation inhibition (from 11.7 ± 0.8 to 5.7 mg GAE/g) compared to those from segments adjacent to the trunk (0 cm). In parallel with what was observed previously for the TPC, interestingly, these results indicate a significant spatial gradient in antioxidant potential along the branch length. Regarding our *Abies alba* twigs (SFT), they are representative of the entire branch length, as all portions—both proximal and distal to the trunk—were included in our study. Therefore, the reported values reflect an average, accounting for higher phenolic contents near the trunk and lower levels toward the branch tips. Also, the lower activity in RAR extracts may reflect sampling from more distal twig portions with reduced phenolic content.

In summary, the antioxidant activity of *Abies alba* twig extracts is not solely dependent on total phenolic content but also on the specific chemical structures and distributions of phenolic compounds, as well as sampling location along the branch. The differential responses observed across FRAP, DPPH, and ORAC assays emphasize the necessity of employing a multifaceted analytical approach when evaluating antioxidant potential in complex plant matrices.

**Figure 2:**
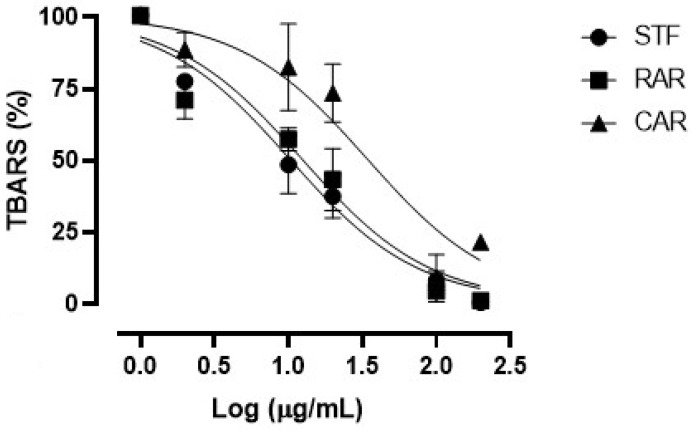
Concentration–response curves of TBARS obtained with the different concentrations (2–200 µg/mL) of the freeze-dried extracts (SFT, RAR and CAR).

Figure 2 shows the concentration–response curves of the TBARS on rat brain assay obtained with the considered extracts. All three extracts showed antioxidant properties in our ex vivo conditions, as demonstrated by their ability to inhibit the formation of MDA. All extracts showed almost full efficacy at the maximum concentration of 200 µg/mL.

Table 6 shows the IC_50_ values calculated for each extract. The SFT extract showed an IC_50_ value equal to 10.20 ± 0.86 µg/mL, similar to that of the RAR extract (12.34 ± 0.92 µg/mL), while the CAR extract (derived from spruce bark) presented the worse potency index (34.11 ± 1.31 µg/mL), significantly lower compared with the other ones. As in the case of the in vitro FRAP antioxidant assay, the relatively higher activity of the SFT extract in the TBARS assay could be due to its higher content in total phenols, which were well-known to be endowed with the ability to quench the oxygen free radicals and delay the formation of the hydroperoxide ion, responsible for lipid peroxidation during the TBARS assay [51].

However, a contribution from other bioactive compounds can not be excluded, including the amino acids; indeed, they exhibit antioxidant activity through various mechanisms, such as scavenging free radicals, chelating metal ions, and modulating antioxidant enzymes [52]. Their antioxidant properties depend significantly on their structure, particularly the side chains (R groups), such as hydroxyl, carboxyl, aromatic units and side chain groups (amino, sulfur, etc.) [53].

Some amino acids can donate electrons or hydrogen atoms to neutralize free radicals like superoxide anion (O^2−^), hydroxyl radical (OH), and peroxyl radicals (ROO). Amino acids with nitrogen or sulfur atoms in their side chains can bind metal ions (like Fe^2+^ and Cu^2+^), which catalyze the Fenton reaction and generate reactive oxygen species (ROS) [54].

Thus, the higher content of amino acids in the SFT extract, as shown in Table 4, could contribute significantly to its antioxidant activity both in vitro (Table 5) and ex vivo (Table 6).

### 2.5. Antimicrobial Activity of the Extracts

The antimicrobial activity of the extracts against selected Gram-negative bacteria (*Escherichia coli* and *Klebsiella aerogenes*) and Gram-positive bacteria (*Enterococcus faecalis* and *Staphylococcus aureus*) was determined by assessing the final growth rate (%).

**Figure 3:**
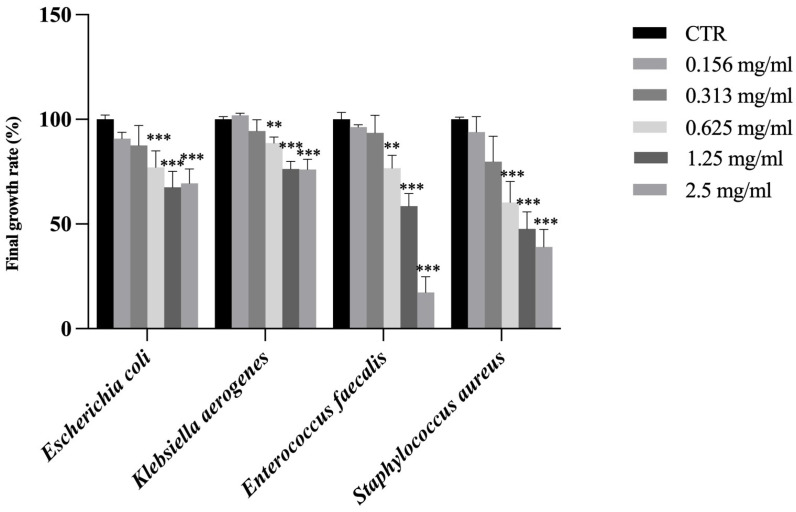
Final growth rate expressed as % of *Escherichia coli*, *Klebsiella aerogenes*, *Enterococcus faecalis* and *Staphylococcus aureus* in the presence of CAR sample at different concentrations (0.156, 0.313, 0.625, 1.25 and 2.5 mg/mL). Results are reported as mean ± standard deviation (*n* = 3). ***, *p* < 0.001; **, *p* < 0.01. CTR is control.

While SFT and RAR extracts showed no antimicrobial activity against these bacteria, CAR extract showed a significant inhibition of the final growth rate at the concentration of 0.625 mg/mL against all bacteria (Figure 3). Among the various types of phenolic compounds contained in trees, some exhibit antibacterial properties: the reason why the CAR extract was the only one to show antimicrobial activity against these Gram-negative and -positive bacteria may be linked to its high content in *trans*-ferulic acid (tFA), almost 5 times higher than in SFT and almost 10 times higher than in RAR. *t*FA exhibits broad-spectrum antimicrobial activity against various bacteria, yeasts, and fungi and showed antimicrobial activity against *Staphylococcus aurous*, *Escherichia coli*, *Enterococcus faecalis*, *Pseudomonas aeruginosa*, *Acinetobacter baumannii*, *Klebsiella pneumonia*, and *Proteus mirabilis* [54] and has been documented to exert antimicrobial effects through disruption of membrane integrity and inhibition of microbial enzymatic systems [55].

These results agree with the remarkable antibacterial activity shown by an HC-based extract of spruce bark from trees growing in Sweden, obtained after a process similar to the one used in this study [26], where the higher activity against both Gram-negative and Gram-positive bacteria shown by the HC-based extract compared to a hot-water extract was ascribed to the higher retention of terpenoids.

Furthermore, a study by Vainio-Kaila et al. [56] demonstrated that methicillin-resistant *Staphylococcus aureus* was susceptible to extracts of *P. Abies*, particularly of heartwood, sapwood, and milled wood lignin of the plant. Another study by Nisca et al. [21] showed that extracts of bark from the same spruce species obtained by different techniques possessed good antioxidant activities, with UAE extracts being the most potent with the respect to MAE, exhibiting low MICs against Gram-positive bacteria, though their efficacy against Gram-negative strains was limited. This result contrasts with the broader-spectrum antimicrobial activity showed by CAR in our study, probably due to the better phenolics extraction capacity of our method.

In a previous study, HC-based extracts of silver fir twigs from trees growing in the same area of the samples collected in this study showed remarkable antibacterial activity against Gram-negative *E. coli*, with the antibacterial activity of the extract, obtained after 10 min of processing time at the temperature of 25 °C, significantly higher than that of the extract obtained after 55 min of processing time at the temperature of 47 °C [3].

This result was ascribed to the higher retention of terpenoids at shorter processing times and lower temperatures. In the same study, the antibacterial activity against Gram-positive *S. aureus* was higher and not significantly dependent on processing conditions.

It is conceivable that the contrasting results obtained in this study for SFT and RAR derive from the long processing time (53 min) and particularly from the high temperature (52 °C) at the end of the process, with the likely consequent removal of terpenoids.

Also, the antimicrobial potential of the CAR extract may reflect the retention of terpenoids, facilitated by the relatively mild extraction conditions employed. Terpenoids are known to compromise bacterial cell membranes and interfere with cellular homeostasis via lipophilic interactions [57]. Similar mechanisms have been observed in HC-derived extracts from coniferous species, where shorter processing times and lower temperatures were associated with higher retention of volatile bioactives and increased antimicrobial activity [33].

Notably, the ability of the CAR extract to inhibit Gram-negative bacteria further supports the potency of its chemical constituents. Gram-negative bacteria typically exhibit greater resistance due to their outer membrane acting as a barrier to many hydrophilic and amphipathic compounds [58]. However, the CAR extract’s chemical composition, rich in low molecular weight phenolics and possibly lipophilic terpenoids, likely allowed for effective membrane penetration and intracellular action across bacterial types.

Furthermore, recent evidence suggests that amino acids co-extracted from lignocellulosic materials can act synergistically with polyphenols by facilitating their uptake through bacterial membranes or by independently targeting metabolic pathways such as protein synthesis and amino acid metabolism [59]. This dual mechanism—membrane disruption by phenolics and metabolic inhibition by amino acids—could enhance the overall bactericidal effect of the CAR extract.

These findings underscore the combined importance of both compound profile and extraction method in determining the antimicrobial efficacy of plant-derived extracts.

### 2.6. Overall Rate of Results with Principal Component Analysis

**Figure 4:**
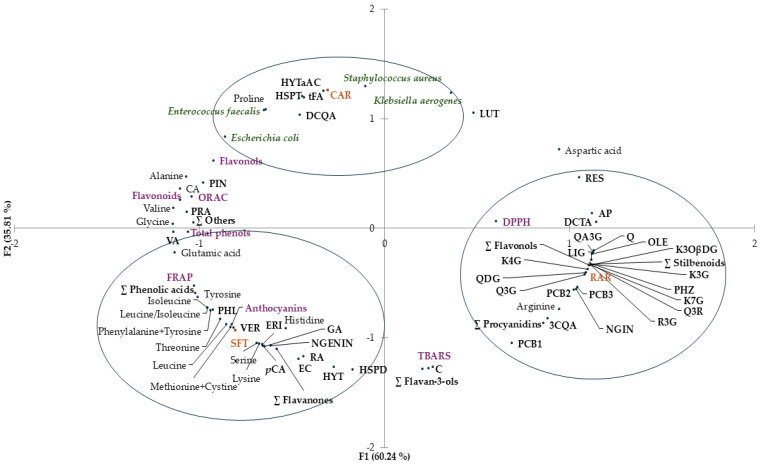
Principal component analysis (PCA) plot with distribution of the variables including total phenols, flavonols, flavonoids, antioxidant activity (DPPH assay, FRAP and ORAC), antimicrobial activity against *Escherichia coli*, *Klebsiella aerogenes*, *Staphylococcus aureus* and *Enterococcus faecalis*, and contents of individual phenolic compounds and free amino acids of freeze-dried extracts (SFT, RAR and CAR). The acronyms at the black dots correspond to the names of the compounds. FRAP, ferric-reducing antioxidant power; ORAC, oxygen radical absorbance capacity; DPPH assay, assay with 2,2-diphenyl-1-picrylhydrazyl radical; PC1, first principal component; PC2, second principal component.

Principal component analysis (PCA) was performed to identify possible relationships between different samples (SFT, RAR and CAR) and variables, i.e., total phenols, phenolic profile, free amino acid content, antioxidant activity, and antimicrobial activity. The distribution of samples (SFT, RAR and CAR) and variables in the PCA plot is shown in Figure 4.

The two first principal components (PC1 and PC2) explained 96.10% of the total variance. A coherent segregation between samples was observed. There was discrimination along PC1 between clustered SFT and CAR and clustered RAR (60.24%).

SFT extract was clustered with anthocyanins, and antioxidant activity evaluated by FRAP, some individual phenolic contents (gallic acid, rosmarinic acid, vanillic acid, *p*-coumaric acid, ∑ phenolic acids, (−)-epicatechin, hesperidin, naringenin, eriodictyol, ∑ flavanones, hydroxytyrosol, verbascoside and phloretin) and some free amino acids (threonine, leucine, isoleucine, leucine/isoleucine, lysine, methionine + cystine, phenylalanine + tyrosine, histidine serine and glutamic acid) were evident.

Antioxidant activity evaluated by DPPH and some individual phenolic contents (chlorogenic acid, chicoric acid, quercetin, quercetin 3-*O*-glucoside, rutin, quercetin 3,4-*O*-diglucoside, quercetagetin 3-*O*-glucoside, kaempferol 7-*O*-glucoside, kaempferol 3-*O*-glucoside, tiliroside, kaempferol 4-*O*-glucoside, ∑ flavonols, naringin, procyanidin B1, procyanidin B2, procyanidin B3, procyanidins, piceid, resveratrol, ∑ stilbenoids, oleuropein, ligstroside, phloridzin and apigenin) and arginine content clustered with RAR extract.

CAR extract was clustered with some individual phenolic contents (protocatechuic acid, caffeic acid, *trans*-ferulic acid, cynarin, hesperetin and hydroxytyrosol a-acetate), with proline content and with the antimicrobial activity against all the selected bacteria (*Escherichia coli*, *Klebsiella aerogenes*, *Enterococcus faecalis* and *Staphylococcus aureus*).

## 3. Materials and Methods

### 3.1. Chemicals and Reagents

All standards and reagents were of analytical grade. Methanol, Folin–Ciocalteu’s reagent, gallic acid, sodium carbonate, acetate buffer, 2,4,6-tri(2-pyridyl)-s-triazine (TPTZ), Trolox, 2,2-diphenyl-1-picrylhydrazyl (DPPH) radical, 1,1,3,3-tetramethoxypropane, ascorbic acid, 2-thiobarbituric acid, iron(III) chloride (FeCl_3_), hydrochloric acid (HCl), sodium hydroxide (NaOH), 2,2′-azobis(2-amidinopropane) dihydrochloride (AAPH), phosphate buffered saline (PBS), and LC-MS/MS standards including gallic acid (GA), protocatechuic acid (PRA), rosmarinic acid (RA), caffeic acid (CA), vanillic acid (VA), *1*, *3*-dicaffeoylquinic acid (DCQA), quercetin 3-*O*-glucoside (Q3G), quercetagetin 3-*O*-glucoside (QA3G), kaempferol 7-*O*-glucoside (K7G), kaempferol 3-*O*-β-D-(6″-*O*-(*E*)-*p*-coumaroyl)glucopyranoside (K3*O*βDG), kaempferol 4-*O*-glucoside (K4G), hesperidin (HSPD), hesperetin (HSPT), naringin (NGIN), naringenin (NGENIN), eriodictyol (ERI), procyanidin B1 (PCB1), procyanidin B2 (PCB2), procyanidin B3 (PCB3), resveratrol 3-*O*-glucoside (R3G), hydroxytyrosol (HYT), hydroxytyrosol a-acetate (HYTaAC), verbascoside (VER), oleuropein (OLE), ligstroside (LIG), pinosterol (PIN), phloretin (PHL), luteolin (LUT) and apigenin (AP) were purchased from Sigma-Aldrich, Inc. (St. Louis, MO, USA). The other chromatographic standards, i.e., 3-*O*-caffeoylquinic acid (3CQA), *p*-coumaric acid (pCA), *trans*-ferulic acid (tFA), cichoric acid (DCTA), quercetin (Q), quercetin 3-*O*-rutinoside (Q3R), quercetin 3,4-*O*-diglucoside (QDG), kaempferol 3-*O*-glucoside (K3G), (+)-catechin (C), (−)-epicatechin (EC), resveratrol (RES) and phloridzin (PHZ) were acquired from Extrasynthese (Genay, France). A certified reference amino acids mix solution was purchased from Supelco (Sigma-Aldrich, Buchs, Switzerland). The mixture contains the following components: Alanine (2.50 mmol/L), Arginine (2.50 mmol/L), Aspartic Acid (2.50 mmol/L), Cystine (1.25 mmol/L), Glutamic Acid (2.50 mmol/L), Glycine (2.50 mmol/L), Histidine (2.50 mmol/L), Isoleucine (2.50 mmol/L), Leucine (2.52 mmol/L), Lysine (2.50 mmol/L), Methionine (2.50 mmol/L), Phenylalanine (2.50 mmol/L), Proline (2.50 mmol/L), Serine (2.50 mmol/L), Threonine (2.50 mmol/L), Tyrosine (2.50 mmol/L), and Valine (2.51 mmol/L) in 0.1 mol/l hydrochloric acid. The bacterial media Mueller Hinton Broth (MHB) and Mueller Hinton Agar (MHA) were purchased from VWR (Radnor, PA, USA).

### 3.2. Origin, Nature and Extraction of Conifer Resources

Spruce (*Picea abies* (L.) H. Karst.) bark (CAR) and twigs (RAR) were collected from high-rise trees in Fiemme Valley, eastern Alps, about 1200 to 1800 m a.s.l. (around 46°15′ N, 11°30′ E). Silver fir (*Abies alba* Mill.) twigs (SFT) were collected in “Teso” forest in Tuscany Apennines, about 1000 to 1100 m a.s.l. (around 44°03′ N; 10°48′ E) from 4 high-rise trees (about 60 years old) and 4 low-rise trees (5–20 years old). Twigs from older trees were collected from lateral branches, no more than 2 m above the soil, while from younger trees, they were collected from the crown branches. Twigs are the smallest and thinnest branches of a tree, serving as the primary sites for the attachment of leaves, needles, or buds. All the fresh biomass samples were stored for 1 week at 4 °C in the dark before milling by means of a Tritone One Electric shredder (Ceccato Olindo srl, Arsego, Italy) and sieving using an electric vibrating sieve (KXY-062, Yuchengtech, Hangzhou, China) equipped with a filter mesh of size 1.5 by 1.5 mm.

The extraction runs were performed immediately after milling and sieving, using an HC pilot plant and water as the only solvent, according to the procedure described in a previous study and using the same equipment [24]. The centrifugal pump afforded a constant discharge of 53.8 m^3^/h. Table 7 shows a few basic features of the extraction tests performed: resource, fresh and dry biomass, solid to liquid ratio, processing time and temperature. The biomass was introduced in the extraction system at the onset of each process. The aqueous extracts collected at the end of the processes were filtered with a 50 µm sieve (stainless steel mesh) and stored in sterile bottles at −20 °C until use.

### 3.3. Extraction Technique

**Figure 5:**
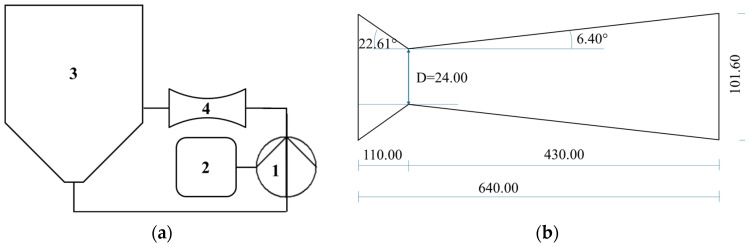
HC device and reactor used in the experiments: (**a**) general layout, with numbers indicating 1—centrifugal pump; 2—electronic control panel; 3—inline tank; 4—Venturi-shaped reactor. (**b**) reactor with throat area of 24 mm.

An HC device with volume capacity of 200 L was used, with a closed hydraulic circuit and a circular Venturi-shaped reactor as the key components, where the liquid–solid mixture was inserted and moved by a centrifugal pump. The pump was an ESHE 50-160/75 (Xylem Water Solutions Italia S.r.l., Lainate, Milan, Italy) model, with nominal power of 7.5 kW, open impeller with a diameter of 174 mm, fixed frequency of 50 Hz and rotation speed of 2900 rpm. All the parts in contact with the circulating mixture were made of food-grade AISI 316 stainless steel. Electricity was the only energy source. The HC device was further described in a previous study [29]. The process was carried out at atmospheric pressure. Power and energy consumption were measured using three-phase digital power meters with power resolution of 1 W and energy resolution of 10 Wh (model D4-Pd, IME, Milan, Italy). Figure 5 shows a schematic representation of the cavitation devices and the Venturi-shaped reactors.

With static HC reactors, such as the Venturi-shaped one used in this study, the simplest representation of cavitation regimes is given by the cavitation number (s), derived from Bernoulli’s law and shown in Equation (1):

(1) $$\sigma =\frac{p_{2}-p_{sat}}{0.5\rho u^{2}}$$

where *p*_2_ is the recovery pressure downstream of the throat (assumed at the level of atmospheric pressure), *p_sat_* is the temperature-dependent saturation vapor pressure of the liquid; *ρ* is the temperature-dependent liquid density; *u* is the flow velocity through the reactor throat, which depends only on pump discharge. Cavitation intensity increases with decreasing cavitation number until the limit of chocked cavitation. In distilled water, the range 0.1 < σ < 1 corresponds to developed cavitation [60]. The geometrical features of the HC reactor and the quantitative method for assessment of σ in the reactor for any process temperature were previously published [27].

Cavitation can also occur around the impeller of a centrifugal pump and can be described by the usual cavitation number, as in Equation (1) [61], whose computation is based on the impeller diameter (174 mm) and rotational speed (2900 rpm), resulting in the velocity of the mixture at the impeller tip (*u* in Equation (1)) of 26.4 m/s, again assuming *p*_2_ at the level of atmospheric pressure.

### 3.4. Extraction Yield

To separate the insoluble solid particles, all the sample extracts were centrifugated at 6000 rpm for 15 min, and the supernatants were freeze-dried. Freeze-drying was performed with a Pascal-LIO 5PDGT (5Pascal, Trezzano sul Naviglio, Italy) at −50 °C and 0.01 mbar, and the obtained final mass was weighed to calculate the total extraction yield. Results were expressed as mg of dry extract per gram of dry biomass.

### 3.5. Phytochemical Characterization of the Conifer Extracts

#### 3.5.1. Extract Preparation

One gram of freeze-dried SFT, RAR or CAR sample was subjected to a double extraction in 10 mL of 80% methanol [62]. Following a 2 h stirring period in the dark, the sample underwent centrifugation at room temperature for 30 min at 4000 rpm. The resulting supernatant was collected, while the pellet obtained was subjected to an additional extraction using another 10 mL of 80% methanol. The supernatants from both extractions were combined to achieve a final concentration of 50 mg/mL and stored at −20 °C until use. The extraction process was conducted in triplicate.

#### 3.5.2. Determination of the Total Phenols, Flavonoids, Flavonols and Anthocyanins

Assay with Folin–Ciocalteu’s reagent was performed to determine the content of total phenols according to the method described by Singleton and co-workers [63].

To measure absorbance, a UV/Vis Lambda 365 spectrophotometer (Perkin Elmer, Waltham, MA, USA) was used. Total phenols content was expressed as mg of gallic acid equivalent per g of extract (mg GAE/g). Flavonoids were quantified using the aluminum chloride colorimetric method and expressed as mg of catechin equivalent per g of extract (mg CE/g) [64]. Flavonols were measured as previously described by Souid et al. [65] and expressed as mg of quercetin equivalent per g of extract (mg QE/g). The different pH spectrophotometric method described by Lee and colleagues [66] was used to quantify total monomeric anthocyanins, expressed as mg of cyanidin-3-glucoside equivalent per g of extract (mg C3GE/g). Results were reported as mean ± standard deviation of triplicates.

#### 3.5.3. Determination of Phenolic and Free Amino Acid Profiles of the Extracts

Liquid chromatography (LC) separation and tandem mass spectrometry (MS/MS) detection of phenolic compounds of SFT, RAR and CAR extracts were performed employing a 5500+ QTrap mass spectrometer with a Turbo V ion-spray source (AB Sciex, Framingham, MA, USA) connected with an Exion LC AC system consisting of two ExionLC AC pumps, autosampler, controller, degasser and tray (Shimadzu, Kyoto, Japan). The extract solutions were injected into a Kinetex Byphenyl column (2.1 × 100 mm, 2.6 µm particle size; Phenomenex, Torrance, CA, USA), and elution was performed in gradient mode using (A) water containing 0.1% (*v*/*v*) formic acid and (B) acetonitrile containing 0.1% (*v*/*v*) formic acid. The gradient program was as follows: 0–10.0 min, 5–95% B; 10.0–12.0 min, 95% B; 12.0–12.1 min, 95–5% B; 12.1–16.0 min, 5% B. The flow rate of the mobile phase was 0.4 mL/min. The MS/MS operation source parameters were as follows: nebulizer gas (GS1), 70 (arbitrary units); turbo gas (GS2), 50 (arbitrary units); curtain gas (CUR), 10 (arbitrary units); temperature, 500 °C; ion spray voltage (IS), −4500 V (for phenolics excluding anthocyanins, negative ion mode) or +5500 V (for anthocyanins, positive ion mode); entrance potential (EP), 10 V; dwell time, 20 ms. Calibration curves for quantitative analysis were built using a standard mix containing all the phenolic compounds at concentrations of 0.5, 1, 2, 4, 8, 16, 32, 64, 128 and 256 ng/mL for gallic acid (GA), protocatechuic acid (PRA), 3-*O*-caffeoylquinic acid (3CQA), rosmarinic acid (RA), caffeic acid (CA), vanillic acid (VA), *p*-coumaric acid (pCA), *trans*-ferulic acid (tFA), cichoric acid (DCTA), 1,3-dicaffeoylquinic acid (DCQA), quercetin (Q), quercetin 3-*O*-glucoside (Q3G), quercetin 3-*O*-rutinoside (Q3R), quercetin 3,4-*O*-diglucoside (QDG), quercetagetin 3-*O*-glucoside (QA3G), kaempferol 7-*O*-glucoside (K7G), kaempferol 3-*O*-glucoside (K3G), kaempferol 3-*O*-β-D-(6″-*O*-(*E*)-*p*-coumaroyl)glucopyranoside (K3*O*βDG), kaempferol 4-*O*-glucoside (K4G), (+)-catechin (C), (−)-epicatechin (EC), hesperidin (HSPD), hesperetin (HSPT), naringin (NGIN), naringenin (NGENIN), eriodictyol (ERI), procyanidin B1 (PCB1), procyanidin B2 (PCB2), procyanidin B3 (PCB3), resveratrol 3-*O*-glucoside (R3G), resveratrol (RES), hydroxytyrosol (HYT), hydroxytyrosol a-acetate (HYTaAC), verbascoside (VER), oleuropein (OLE), ligstroside (LIG), pinosterol (PIN), phloridzin (PHZ), phloretin (PHL), luteolin (LUT) and apigenin (AP). Amino acid analysis was carried out using a Restek Raptor Polar X column (2.1 × 100 mm, 2.7 µm particle size; Restek Corporation, Bellefonte, PA, USA). Elution was performed in gradient mode using the same solvents, and the program was 0–2 min, 4% B; 2–10 min, 4–70% B; 10.0–10.1 min, 70–95% B; 10.1–13 min, 95% B. The MS/MS operation source parameters were as follows: nebulizer gas (GS1), 40 (arbitrary units); turbo gas (GS2), 60 (arbitrary units); curtain gas (CUR), 30 (arbitrary units); temperature, 400 °C; ion spray voltage (IS), +5500 V. Nitrogen was used as collision gas. Compound-dependent parameters including de-clustering potential (DP), collision energy (CE), and collision cell exit potential (CXP) were adjusted for the specific selected reaction monitoring (SRM) transition for each compound. An information-dependent acquisition (IDA) approach, with one selected reaction monitoring (SRM) transition per component for quantification and as a survey scan and MS-MS enhanced product ion (EPI) spectrum acquisition, was used for qualitative identification.

Calibration curves for quantitation of amino acids were built using the reference standard mix diluted to 0.01, 0.02, 0.04, 0.08, 0.16, 0.32, 0.64, and 1.28 mmol/L for Alanine, Arginine, Aspartic Acid, Glutamic Acid, Glycine, Histidine, Isoleucine, Leucine, Lysine, Methionine, Phenylalanine, Proline, Serine, Threonine, Tyrosine and Valine, and to 0.005, 0.01, 0.02, 0.04, 0.08, 0.16, 0.32, and 0.64 mmol/L for Cystine. Results were reported as mean ± standard deviation of triplicates.

### 3.6. Evaluation of Biological Properties of Conifer Extracts

#### 3.6.1. In Vitro Antioxidant Activity Assays

The antioxidant activity of SFT, RAR and CAR extracts was explored as FRAP, DPPH scavenging activity and ORAC. The antioxidant capacity of sample extracts to reduce ferric iron (Fe^3+^) to ferrous iron (Fe^2+^) was determined by the FRAP assay, as previously reported [67]. Briefly, a solution containing 300 mM acetate buffer (pH 3.6), 10 mM 2,4,6-Tri(2-pyridyl)-s-triazine (TPTZ) in 40 mM HCl, and 20 mM FeCl_3_·6H_2_O was added to each extract (85 μL) at a concentration of 50 mg/mL diluted in 80% methanol. Following 30 min incubation at room temperature, the absorbance was measured at 593 nm using a Perkin-Elmer Lambda 365 spectrophotometer (PerkinElmer Italia, Milano, Italy). The results were calculated based on the standard curve for Trolox and expressed as mg of Trolox equivalent (TE) per g of extract.

The radical scavenging activity of the sample extracts was determined using the 2,2-diphenyl-1-picrylhydrazyl (DPPH) assay, as described by Boudjou et al. [68], with some modifications. Briefly, 400 μL of each extract (50 mg/mL diluted in 80% methanol) was added to 3600 μL of DPPH solution (44 μM in 80% methanol) and incubated for 40 min in the dark at room temperature. The absorbance was recorded at 517 nm using a Perkin-Elmer Lambda 365 spectrophotometer (Perkin ElmerItalia, Milano, Italy). Trolox was used as standard, and results were expressed as mg of TE per g of extract.

The oxygen radical absorbance capacity (ORAC) of sample extracts was determined as described by Grande and co-workers [69], with some modifications. An aliquot of 100 µL of sample extract (50 mg/mL diluted in 80% methanol) or 50 µM Trolox was mixed with 800 µL of 40 nM fluorescein sodium salt in 75 mM phosphate buffer (pH 7.4) and with 100 µL of 400 mM AAPH. Fluorescein was used as a probe, and AAPH was used as a peroxyl radical generator. Fluorescein fluorescence decay was read at λex 485 nm and λem 510 nm. AAPH was used as a peroxyl radical generator, fluorescein as the probe, and Trolox as the standard. Results were expressed as mg of TE per g of extract and were reported as mean ± standard deviation of triplicates.

#### 3.6.2. Ex Vivo Antioxidant Activity Assay

##### Animals

Male Wistar rats aged 12–15 weeks and weighing between 300 and 400 g were used. The animals were housed in cages, with freedom of movement, supplied with water and food, and exposed to light/dark cycles of 12 h each, at 22 °C. The study was carried out in line with EU legislation (EEC Directive 63/2010) and Italian legislation (Legislative Decree No. 26/2014) on the protection of animals used for scientific purposes. Formal approval to conduct the described experiments was obtained from the Animal Subjects Review Board of the University of Pisa, where we discussed it and obtained the following codex: DB173.N.IXS. (protocol number 46/2023, 24 October 2023).

The animals were euthanized following anesthesia with isoflurane (R584S Small Animal Aesthesia Machine, Shenzhen RWD Life Science and Technology Co., Ltd., San Diego, CA, USA). Then, the brains were removed and immediately chopped and homogenized in 10% (*w*/*v*) phosphate buffer, pH 7.4, using an Ultra-Turrax homogenizer (IKA, T-18 Basic, IKA-Werke GmbH & Co., Staufen, Germany).

##### Thiobarbituric Acid-Reactive Substance (TBARS) Assay

The TBARS assay was performed according to a previously published protocol [70]. All used analytical-grade reagents and solvents were purchased from Sigma Aldrich (St. Louis, MO, USA) and used without any purification. The stock solutions of SFT/CAR/RAR extracts (50 mg/mL) were diluted at different concentrations with phosphate buffer pH 7.4. Specifically, the extracts were tested in the range of concentrations between 2–200 µg/mL. The experiment was carried out in falcon tubes, where the rat brain homogenate was treated with FeCl_3_ (20 µM) and ascorbic acid (100 µM) in the presence or absence of the studied extract. The solutions were heated at 37 °C and stirred for 30 min. Then, thiobarbituric acid and 25% *v*/*v* HCl were added, and the tubes were heated at 100 °C for 10 min. Afterward, the samples were cooled on ice. The water solutions were extracted with n-butanol, and the organic part was aliquoted into 96-well plates. The absorbance at 532 nm, was recorded using a SPECTROstarNano (220–1000 nm) UV–Vis spectrophotometer (Ortenberg, Germany). The TBARS was reported as nmoles of malondialdehyde (MDA)/10 mg of rat brain tissue, by interpolation with the standard curve of 1,1,3,3-tetramethoxypropane. Each result was obtained with brain of at least 3 different animals. All data were expressed as mean ± SEM. The oxidation inhibition rate at the extract concentration of 100 µg/mL was used for the PCA.

#### 3.6.3. Determination of the Antimicrobial Activity

The bacterial strains were supplied by the German Collection of Microorganisms and Cell Cultures GmbH (DSMZ; Braunschweig, Germany). The antimicrobial activity of SFT, RAR and CAR extracts was analyzed against two Gram-negative bacteria, specifically *Escherichia coli* (DSM 1103) and *Klebsiella aerogenes* (DSM 30053), and two Gram-positive bacteria, *Enterococcus faecalis* (DSM 2570) and *Staphylococcus aureus* (DSM 25693). The effect of extracts on the growth of selected bacteria was determined according to a procedure reported in our earlier study [71], with some modifications. The bacteria were cultured in MHB at 37 °C for 16 h and diluted to match the turbidity of 0.5 McFarland standard. Then, bacterial suspensions containing about 1–5 × 10^5^ CFU/mL (50 µL) MHB (100 µL) and sample extract solutions with concentrations of 0.313, 0.625, 1.25 and 2.5 mg/mL (100 µL) were pipetted into a 96-well plate. Concurrently, a control with water instead of extract solution was added into the plate. The optical density (O.D.) was measured at 630 nm using a microplate reader (Eti-System fast reader Sorin Biomedica, Modena, Italy) after plate incubation at 37 °C for 24 h. Results were expressed as final growth rate (%) with reference to the final density of the bacteria incubated with or without extracts, calculated according to Equation (2).

Final growth rate (%) = (O.D._sample_/O.D._control_) × 100 (2)

where O.D._sample_ is the optical density of the measurement with extract and O.D._control_ is the optical density of the control without extract.

In addition, the inhibition rate was calculated according to Equation (3). This parameter was used in the PCA.

Inhibition rate (%) = (100 − O.D._sample_/O.D._control_) × 100 (3)

where O.D._sample_ is the optical density of the measurement with extract and O.D._control_ is the optical density of the control without extract.

### 3.7. Statistical Analysis

The statistically significant (*p* < 0.05) differences between extracts in phytochemical composition, in vitro and ex vivo antioxidant and antimicrobial activity were valued with analysis of variance (ANOVA) (GraphPad Prism 8.0).

PCA was performed to analyze multivariate data and to characterize and differentiate SFT, RAR, and CAR extracts in relation to the variables (total phenols, flavonols, flavonoids, in vitro and ex vivo antioxidant activity, antimicrobial activity against *Escherichia coli*, *Klebsiella aerogenes*, *Staphylococcus aureus* and *Enterococcus faecalis*, and contents of individual phenolic compounds and free amino acids). Analyses were performed using XLSTAT software (version 2019).

## 4. Conclusions

This study investigated the exploitation of conifer by-products using HC as an emerging extraction technique, through the characterization of the obtained extracts in terms of phenolic compounds, free amino acids and antioxidant and antimicrobial capacity, as objective premises for possible applications.

Specifically, SFT extract was mainly characterized by a high quantity of anthocyanins, ∑ phenolic acids, ∑ flavanones and all free essential amino acids plus some dispensable free amino acids (glycine and glutamic acid) and the highest FRAP activity. The RAR extract showed higher content of ∑ flavonols, ∑ procyanidins and ∑ stilbenoids, higher DPPH scavenging activity and higher content of aspartic acid and arginine. Finally, the CAR extract showed the most effective antimicrobial activity against Gram-negative (*Escherichia coli* and *Klebsiella aerogenes*) and Gram-positive (*Enterococcus faecalis* and *Staphylococcus aureus*) bacteria and the highest content of protocatechuic acid, caffeic acid, *trans*-ferulic acid, hesperetin, hydroxytyrosol α-acetate, pinosterol and apogenin, as regards phenolic compounds, and alanine and proline, as regards free amino acids.

Notably, the SFT and RAR extracts exhibited significantly higher activity against lipid peroxidation in the ex vivo TBARS essay, compared to the CAR extract, confirming the differentiation of the properties among the extracts.

This study demonstrated that softwood bark and twig by-products, particularly those from *Picea abies* and *Abies alba*, are valuable sources of bioactive compounds. Using an energy-efficient HC extraction method, diverse phenolic compounds and free amino acids were successfully identified in the extracts. Notably, PCA confirmed the effect of the sample on phenolic compounds, free amino acids and antioxidant and antibacterial activities. Indeed, although the extracts exhibited notable antioxidant capacity, the SFT extract showed the highest activity, and the CAR extract uniquely displayed antimicrobial effects. These findings highlight the untapped potential of coniferous biomass by-products for applications in the pharmaceutical, nutraceutical, and food industries, as well as warranting further in vivo and eventually clinical investigations.

As pointed out in a recent study [24], the industrialization of the HC extraction method is technically feasible, while its economic sustainability is conditioned on the use of a sufficient biomass concentration, as most of the energy is consumed in the water removal steps. For example, the use of a dry biomass to water ratio of 1:8 for spruce bark allows assessing a specific energy consumption in a full-scale setup of around 60 kWh per kg of dry extract [24], which can help in assessing the sustainability of projects aimed at manufacturing dry extracts from conifer byproducts.

## Figures and Tables

**Table 1:** Total extraction yield of samples (SFT, RAR and CAR) extracted using HC.

Sample	Total Extraction Yield (mg/g dw)
SFT	135.91 ^B^ ± 1.01
RAR	164.55 ^A^ ± 6.15
CAR	141.97 ^B^ ± 8.47

Results are expressed as mean ± standard deviation of three replicates. Letters A and B show statistically significant differences.

**Table 2:** Bioactive compounds of freeze-dried extracts (SFT, RAR and CAR).

Sample	Total Phenols (mg GAE/g)	Flavonoids (mg CE/g)	Flavonols (mg QE/g)	Anthocyanins (µg C3GE/g)
SFT	108.11 ^A^ ± 7.82	89.18 ^A^ ± 5.60	6.08 ^A^ ± 0.84	8.23 ^A^ ± 0.92
RAR	75.71 ^B^ ± 5.96	59.43 ^B^ ± 1.56	4.16 ^B^ ± 0.48	0.35 ^B^ ± 0.12
CAR	98.65 ^A^ ± 8.55	87.52 ^A^ ± 3.23	6.84 ^A^ ± 0.37	2.10 ^B^ ± 0.94

Results are expressed as mean ± standard deviation of three replicates. Letters A and B show statistically significant differences for each column of data.

**Table 3:** Content of individual phenolic compounds (µg/g) of freeze-dried extracts (SFT, RAR and CAR).

Compound Name	Acronym	SFT	RAR	CAR
Gallic acid	GA	274.30 ^A^ ± 5.52	74.37 ^B^ ± 0.62	27.55 ^C^ ± 0.05
Protocatechuic acid	PRA	357.22 ^A^ ± 2.28	300.91 ^B^ ± 20.74	348.52 ^A^ ± 1.38
3-*O*-Caffeoylquinic acid (Chlorogenic acid)	3CQA	0.65 ^B^ ± 0.04	1.08 ^A^ ± 0.02	0.34 ^C^ ± 0.07
Rosmarinic acid	RA	0.14 ^A^ ± 0.01	0.07 ^B^ ± 0.03	0.02 ^C^ ± 0.01
Caffeic acid	CA	19.98 ^B^ ± 0.01	6.37 ^C^ ± 0.02	20.40 ^A^ ± 0.16
Vanillic acid	VA	82.09 ^A^ ± 1.87	40.44 ^C^ ± 1.04	70.25 ^B^ ± 2.88
*p*-Coumaric acid	*p* CA	142.21 ^A^ ± 2.96	36.23 ^B^ ± 0.66	13.15 ^C^ ± 0.36
*trans*-Ferulic acid	tFA	31.68 ^B^ ± 1.83	15.20 ^C^ ± 0.13	135.33 ^A^ ± 2.47
2,3-Dicaffeoyl-tartaric acid (Chicoric acid)	DCTA	0.11 ^C^ ± 0.00	4.58 ^A^ ± 0.35	1.47 ^B^ ± 0.10
1,3-Dicaffeoylquinic *acid* (Cynarin)	DCQA	0.01 ^B^ ± 0.01	n.d. ^B^	0.03 ^A^ ± 0.01
∑ Phenolic Acids		908.39 ^A^ ± 0.31	479.26 ^C^ ± 21.22	617.06 ^B^ ± 7.37
Quercetin	Q	0.06 ^B^ ± 0.03	0.58 ^A^ ± 0.00	0.11 ^B^ ± 0.03
Quercetin 3-*O*-glucoside	Q3G	0.83 ^B^ ± 0.07	4.33 ^A^ ± 0.23	0.49 ^B^ ± 0.01
Quercetin 3-*O*-rutinoside (Rutin)	Q3R	0.03 ^B^ ± 0.01	16.49 ^A^ ± 0.57	0.07 ^B^ ± 0.00
Quercetin 3,4-*O*-diglucoside	QDG	1.23 ^B^ ± 0.02	16.30 ^A^ ± 1.19	0.12 ^B^ ± 0.00
Quercetagetin 3-*O*-glucoside	QA3G	0.49 ^B^ ± 0.06	5.59 ^A^ ± 0.24	0.93 ^B^ ± 0.07
Kaempferol 7-*O*-glucoside	K7G	0.13 ^B^ ± 0.01	17.69 ^A^ ± 0.23	0.05 ^B^ ± 0.01
Kaempferol 3-*O*-glucoside	K3G	0.12 ^B^ ± 0.00	107.28 ^A^ ± 1.38	0.13 ^B^ ± 0.01
Kaempferol 3-*O*-β-D-(6″-*O*-(E)-p-coumaroyl)glucopyranoside (Tiliroside)	K3OβDG	0.07 ^B^ ± 0.01	1.24 ^A^ ± 0.10	0.06 ^B^ ± 0.01
Kaempferol 4-*O*-glucoside	K4G	1.50 ^B^ ± 0.05	34.91 ^A^ ± 1.45	0.03 ^B^ ± 0.01
∑ Flavonols		4.45 ^B^ ± 0.15	204.41 ^A^ ± 1.23	1.97 ^C^ ± 0.13
(+)-Catechin	C	97.14 ^A^ ± 1.45	91.57 ^B^ ± 0.52	12.67 ^C^ ± 0.23
(−)-Epicatechin	EC	7.13 ^A^ ± 0.10	3.81 ^B^ ± 0.11	1.96 ^C^ ± 0.04
∑ Flavan-3-ols		104.27 ^A^ ± 1.56	95.39 ^B^ ± 0.63	14.63 ^C^ ± 0.27
Hesperidin	HSPD	0.23 ^A^ ± 0.01	0.17 ^B^ ± 0.01	0.09 ^C^ ± 0.00
Hesperetin	HSPT	0.02 ^B^ ± 0.00	0.00 ^B^ ± 0.00	0.12 ^A^ ± 0.02
Naringin	NGIN	0.12 ^B^ ± 0.00	0.29 ^A^ ± 0.00	0.08 ^C^ ± 0.01
Naringenin	NGENIN	3.42 ^A^ ± 0.56	1.00 ^B^± 0.19	0.38 ^B^ ± 0.04
Eriodictyol	ERI	1.09 ^A^ ± 0.05	0.45 ^B^ ± 0.05	0.32 ^C^ ± 0.05
∑ Flavanones		4.88 ^A^ ± 0.61	1.91 ^B^ ± 0.25	0.98 ^B^ ± 0.10
Procyanidin B1	PCB1	54.65 ^B^ ± 0.11	78.15 ^A^ ± 0.54	14.15 ^C^ ± 0.41
Procyanidin B2	PCB2	10.92 ^B^ ± 0.86	35.69 ^A^ ± 5.20	4.60 ^B^ ± 0.91
Procyanidin B3	PCB3	4.31 ^B^ ± 0.27	14.96 ^A^ ± 1.26	1.98 ^C^ ± 0.37
∑ Procyanidins		69.88 ^B^ ± 1.24	128.80 ^A^ ± 6.99	20.73 ^C^ ± 1.69
Resveratrol 3-*O*-glucoside (Piceid)	R3G	0.10 ^B^ ± 0.01	68.06 ^A^ ± 2.04	0.02 ^B^ ± 0.00
Resveratrol	RES	0.17 ^C^ ± 0.04	0.56 ^A^ ± 0.05	0.40 ^B^ ± 0.04
∑ Stilbenoids		0.27 ^C^ ± 0.05	68.62 ^A^ ± 2.09	0.42 ^B^ ± 0.03
Hydroxytyrosol	HYT	4.91 ^A^ ± 0.13	3.20 ^B^ ± 0.00	1.41 ^C^ ± 0.04
Hydroxytyrosol a-acetate	HYTaAC	0.03 ^B^ ± 0.00	0.01 ^B^ ± 0.00	0.63 ^A^ ± 0.02
Verbascoside	VER	0.33 ^A^ ± 0.03	0.06 ^B^ ± 0.01	0.07 ^B^ ± 0.02
Oleuropein	OLE	0.08 ^B^ ± 0.01	0.13 ^A^ ± 0.00	0.08 ^B^ ± 0.00
Ligstroside	LIG	0.01 ^B^ ± 0.00	0.12 ^A^ ± 0.00	0.02 ^B^ ± 0.00
Pinosterol	PIN	37.76 ^A^ ± 0.65	28.78 ^B^ ± 2.39	39.06 ^A^ ± 3.78
Phloridzin	PHZ	0.02 ^B^ ± 0.00	1.56 ^A^ ± 0.00	0.03 ^B^± 0.01
Phloretin	PHL	0.20 ^A^± 0.02	0.01 ^B^ ± 0.01	0.04 ^B^ ± 0.01
Luteolin	LUT	0.02 ^B^ ± 0.00	0.06 ^A^ ± 0.00	0.08 ^A^ ± 0.03
Apigenin	AP	0.01 ^C^ ± 0.00	0.05 ^A^ ± 0.00	0.02 ^B^ ± 0.01
∑ Others		43.35 ^A^ ± 0.47	33.95 ^B^ ± 2.38	41.44 ^A^ ± 3.64

Results are expressed as mean ± standard deviation of three replicates. n.d., not detected. Letters A, B and C show statistically significant differences for each row of data.

**Table 4:** Content of individual free amino acids (ng/g) of freeze-dried extracts (SFT, RAR and CAR).

Amino Acid	SFT	RAR	CAR
	Essential Amino Acids		
Valine	112.51 ^A^ ± 0.62	9.38 ^C^ ± 0.12	99.66 ^B^ ± 0.61
Threonine	141.49 ^A^ ± 6.07	11.83 ^C^ ± 0.19	26.38 ^B^ ± 0.55
Leucine	9013.71 ^A^ ± 381.10	37.30 ^C^ ± 0.82	624.03 ^B^ ± 38.24
Isoleucine	141.90 ^A^ ± 2.70	8.14 ^C^ ± 0.05	36.47 ^B^ ± 1.14
Leucine/Isoleucine	63.50 ^A^ ± 1.48	4.58 ^C^ ± 0.07	17.10 ^B^ ± 0.51
Lysine	40.99 ^A^ ± 0.89	10.01 ^B^ ± 0.30	4.27 ^C^ ± 0.03
Methionine + Cystine	15.95 ^A^ ± 0.43	0.43 ^B^ ± 0.03	1.03 ^B^ ± 0.05
Phenylalanine + Tyrosine	328.87 ^A^ ± 5.16	23.62 ^C^ ± 0.50	81.99 ^B^ ± 2.44
Histidine	7.96 ^A^ ± 1.65	5.75 ^A,B^ ± 0.22	5.15 ^B^ ± 0.23
	Dispensable Amino Acids		
Glycine	12.24 ^A^ ± 0.03	5.30 ^C^ ± 0.18	10.64 ^B^ ± 0.38
Alanine	86.33 ^B^ ± 2.76	23.54 ^C^ ± 0.04	95.20 ^A^ ± 0.79
Serine	22.08 ^A^ ± 0.48	5.90 ^B^ ± 0.04	3.17 ^C^ ± 0.00
Proline	50.15 ^B^ ± 0.07	37.17 ^C^ ± 0.38	77.27 ^A^ ± 0.42
Aspartic acid	27.21 ^C^ ± 0.26	39.67 ^A^ ± 1.02	37.03 ^B^ ± 1.16
Glutamic acid	165.84 ^A^ ± 0.31	51.05 ^C^ ± 0.66	118.52 ^B^ ± 0.48
Arginine	29.39 ^B^ ± 0.35	83.40 ^A^ ± 0.14	0.52 ^C^ ± 0.05

Results are expressed as mean ± standard deviation of three replicates. Letters A, B and C show statistically significant differences for each row of data.

**Table 5:** In vitro antioxidant activity of freeze-dried extracts (SFT, RAR and CAR).

Sample	FRAP (mg TE/g)	DPPH (mg TE/g)	ORAC (mg TE/g)
SFT	175.65 ^A^ ± 11.70	121.49 ± 5.51	687.57 ^A^ ± 71.34
RAR	104.71 ^C^ ± 3.11	127.23 ± 3.66	507.26 ^B^ ± 5.47
CAR	130.27 ^B^ ± 6.35	123.51 ± 5.37	683.31 ^A^ ± 16.43

Results are expressed as mean ± standard deviation of three replicates. Letters A, B and C show statistically significant differences for each column of data.

**Table 6:** IC_50_ ± SEM values of the TBARS assay calculated for each freeze-dried extract (SFT, RAR and CAR).

Sample	IC_50_ ± SEM (µg/mL)
SFT	10.20 ^B^ ± 0.86
RAR	12.34 ^B^ ± 0.92
CAR	34.11 ^A^ ± 1.31

Results are expressed as mean ± standard error of three replicates. Letters A and B show statistically significant differences.

**Table 7:** Basic features of the extraction tests.

Test ID	Resource	Fresh Biomass (kg)	Dry Biomass (kg)	Concentration (Dry Biomass to Water) ^a^	Time (min)	Temperature (°C)
SFT	Silver fir twigs	5.6	4.4	1:34	53	22.0–52.0
RAR	Spruce twigs	20.1	16.1	1:9	54	20.0–52.5
CAR	Spruce bark	23.7	19.0	1:8	58	20.0–55.5

^a^ The ratio does not include the water contained in the fresh biomass.

## Data Availability

Dataset available on request from the authors.
